# DeepVISP: Deep Learning for Virus Site Integration Prediction and Motif Discovery

**DOI:** 10.1002/advs.202004958

**Published:** 2021-03-08

**Authors:** Haodong Xu, Peilin Jia, Zhongming Zhao

**Affiliations:** ^1^ Center for Precision Health School of Biomedical Informatics The University of Texas Health Science Center at Houston (UTHealth) Houston TX 77030 USA; ^2^ MD Anderson Cancer Center UTHealth Graduate School of Biomedical Sciences Houston TX 77030 USA; ^3^ Department of Biomedical Informatics Vanderbilt University Medical Center Nashville TN 37203 USA

**Keywords:** cancer, deep learning, EBV, HBV, HPV, viruses

## Abstract

Approximately 15% of human cancers are estimated to be attributed to viruses. Virus sequences can be integrated into the host genome, leading to genomic instability and carcinogenesis. Here, a new deep convolutional neural network (CNN) model is developed with attention architecture, namely DeepVISP, for accurately predicting oncogenic virus integration sites (VISs) in the human genome. Using the curated benchmark integration data of three viruses, hepatitis B virus (HBV), human herpesvirus (HPV), and Epstein‐Barr virus (EBV), DeepVISP achieves high accuracy and robust performance for all three viruses through automatically learning informative features and essential genomic positions only from the DNA sequences. In comparison, DeepVISP outperforms conventional machine learning methods by 8.43–34.33% measured by area under curve (AUC) value enhancement in three viruses. Moreover, DeepVISP can decode *cis*‐regulatory factors that are potentially involved in virus integration and tumorigenesis, such as HOXB7, IKZF1, and LHX6. These findings are supported by multiple lines of evidence in literature. The clustering analysis of the informative motifs reveales that the representative k‐mers in clusters could help guide virus recognition of the host genes. A user‐friendly web server is developed for predicting putative oncogenic VISs in the human genome using DeepVISP.

## Graphical abstract

Approximately 15% of human cancers are estimated to be attributed to viruses. This study presents a new deep learning model with attention architecture, namely DeepVISP, for accurately predicting oncogenic virus integration sites (VISs) in the human genome. Moreover, DeepVISP decodes several cis‐regulatory factors that are potentially involved in virus integration and tumorigenesis.

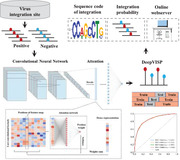

## 1 Introduction

Viral infection has been frequently reported in human diseases including cancer.^[^ ^1^ ^]^ The molecular mechanisms of viral oncogenesis are complicated. To date, numerous mechanisms have been reported, such as chronic inflammation, cell cycle dysregulation, interference with cellular DNA repair mechanisms resulting in genome instability and disruption of host genetic and epigenetic integrity, among others.^[^ ^2^ ^]^ Epstein‐Barr virus (EBV) is the first human virus to be classified as carcinogenic in 2004.^[^ ^3^ ^]^ Since then, studies have reported EBV infection in a variety of human cancers, including Burkitt lymphoma, Hodgkin lymphomas, NK/T cell lymphomas, and many subtypes of gastric carcinomas.^[^ ^3^, ^4^ ^]^ Persistent hepatitis B virus (HBV) infection associated with chronic inflammation may result in chronic liver diseases, progression to cirrhosis and subsequent development of hepatocellular carcinoma (HCC), which is the fifth or sixth most prevalent cancer today.^[^ ^5^ ^]^ Human herpesvirus (HPV) infection, specifically its subset of mucosotropic HPVs (i.e., “high‐risk” HPVs), is associated with more than 99% of human cervical carcinoma, squamous‐cell carcinoma and head and neck squamous cell carcinoma (HNSC)^[^ ^1^, ^6^ ^]^. For instance, the integration of HBV in *SERCA1* gene disrupts the calcium homeostasis of the endoplasmic reticulum and induces apoptosis.^[^ ^7^ ^]^ In another example, insertion of HPV E7 gene into tumor suppressor gene *RB1* could activate the infected quiescent cells to become a proliferative state, which might induce viral genome replication.^[^ ^8^ ^]^ Moreover, the insertion of EBV into some tumor suppressor genes or inflammation‐related genes (e.g., *TNFAIP3*, *CDK15*, and *PARK2*) could disrupt gene function and, accordingly, dysregulate many biological pathways leading to cancer.^[^ ^9^ ^]^ In summary, ≈15% of human cancer cases are attributed to oncogenic viruses.^[^ ^10^ ^]^ This calls for novel methods and computational tools for better detecting viruses and their integration sites in the host genome.

Various approaches have been applied to detect virus infection in the host cells, and if infected, whether viruses are integrated into the host genome and where those viral integration sites (VISs) are located. Traditional approaches include fluorescence in situ hybridization (FISH), amplification of papillomavirus oncogenic transcript assay, and various polymerase chain reaction (PCR) methods. Recently, next‐generation sequencing (NGS) has become a powerful method for detecting virus infection and identify VISs or other types of mutations.^[^ ^11^ ^]^ For example, using high‐throughput sequencing, Zhao et al. reported more than 4220 HBV integration events in tumor and adjacent non‐tumor samples from 426 patients with HCC and systematically explored genomic and oncogenic preferences for HBV integration.^[^ ^12^ ^]^ Moreover, Hu et al. detected 3667 HPV integration events by conducting whole genome sequencing and high‐throughput viral detection. They further identified the clustered genomic hot spots and a potential mechanism for integration by microhomology.^[^ ^13^ ^]^ Computational tools have been developed for the detection of viruses and VISs in the host genomes from genome sequencing data.^[^ ^14^ ^]^ Subsequently, many VISs with experimental evidence have been reported in the human genomes. We have recently manually curated these VISs, including their flanking sequences, and released the Viral Integration Site DataBase (VISDB).^[^ ^15^ ^]^ The current version of VISDB contains a total of 77632 VISs of five DNA viruses and four RNA retroviruses. Our curated VIS data provide benchmarks for the development of computational methods to predict potential viral integration sites in the host (human) genome.

**Figure 1:**
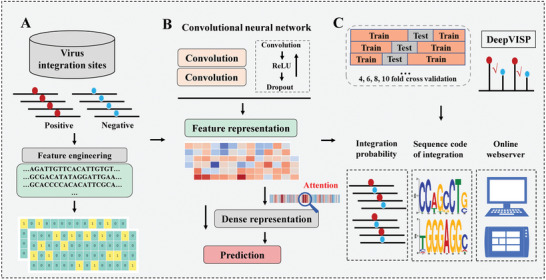
DeepVISP overview. It includes A) benchmark datasets and feature processing, B) deep learning model construction, and C) evaluation, features, and website of DeepVISP.

Deep learning has recently been demonstrated powerful for mining large but complex biomedical data, including natural language processing and imaging data.^[^ ^16^ ^]^ In this study, we developed a deep convolutional neural networks (CNN) model with attention architecture for accurately predicting oncogenic VISs in the human genomes through automatically learning informatic features and essential genomic positions from primary DNA sequences. We called our model DeepVISP (**Deep** learning for **V**iral **I**ntegration **S**ite **P**rediction) (**Figure** **1**). We first compiled three benchmark datasets from our VISDB database, containing 20588, 5118 and 1112 experimentally validated VISs for HBV, HPV and EBV, respectively. These three viruses contained the largest number of VISs in VISDB. We examined the target genes and other genome characteristics of these VISs. We implemented six other machine‐learning models, i.e., AdaBoost (AB), Decision Trees (DT), K‐Nearest Neighbors (KNN), Logistic Regression (LR), Random Forests (RF) and Support Vector Machine (SVM), and compared the DeepVISP with these conventional machine‐learning algorithms across three sets of baseline data. DeepVISP consistently had average Area Under Curve (AUC) values above 0.8 using multiple fold cross‐validations (CVs) among all three types of viruses in the training and independent datasets, demonstrating its robustness. In comparison, DeepVISP outperforms conventional machine learning methods by increasing the AUC value by a range of 8.43% to 34.33% in these three viruses. In addition, CNN is effective on motif discovery through extracting important sequence features. When we applied DeepVISP to decode informative motifs in different types of oncogenic VISs, some motifs could match DNA‐binding transcription factors that were previously reported to be involved in virus insertion or tumorigenesis, such as HOXB7,^[^ ^17^ ^]^ LHX6,^[^ ^18^ ^]^ and IKZF1.^[^ ^19^ ^]^ Our results provided some novel insights into cis‐regulatory factors in the human genome for future study of their oncogenic viral integration and clinical outcome. A deeper view of these motifs suggested that dominant k‐mers in the clusters might play a crucial guidance role in viral recognition of host genes. A user‐friendly online tool for oncogenic VIS prediction is publicly available at https://bioinfo.uth.edu/DeepVISP.

## 2 Results

### 2.1 The Landscape of Oncogenic Viral Integration in the Human Genome

**Figure 2:**
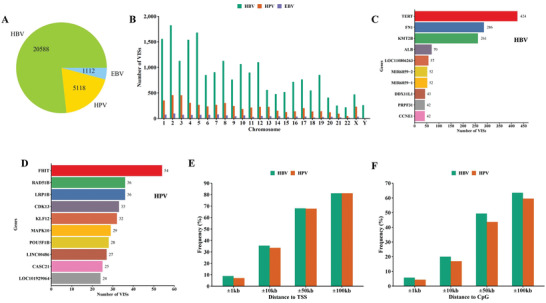
Summary of viral integration site (VIS) data for three viruses. A) Number of VISs of each virus. B) Distribution of VISs of each virus across the human chromosomes. C) The top 10 genes harboring the largest number of HBV integration sites in the human genome. D) The top 10 genes harboring the largest number of HPV integration sties in the human genome. E) Frequency of HBV and HPV VISs falling into the transcript start site (TSS) regions. F) Frequency of HBV and HPV VISs falling into the CpG island (CGI) regions.

In this study, three oncogenic viruses were compiled from the VISDB database, which was manually extracted and curated from the literature and other publicly available resources, containing 20588 HBV, 5118 HPV, and 1112 EBV VISs, respectively (Tables S1–S3, Supporting Information; **Figure** **2**A). Figure 2B shows the distribution of these oncogenic VISs across the human chromosomes. For HBV, the top three chromosomes with the largest number of VISs were chromosomes 2 (8.85%), 5 (8.16%), and 1 (7.56%). For HPV, the number of VISs varied among the 24 human chromosomes, with the largest number of VISs occurring on chromosomes 2 (8.89%), 3 (8.87%), and 1 (6.86%). EBV had fewer VISs than HBV and HPV. Its top three chromosomes with the largest number of VISs were chromosomes 2 (8.72%), 7 (7.10%), and 1 (6.74%). We next examined the target genes of VISs. Figure 2C,D showed the top 10 genes with the largest number of VISs for HBV and HPV. In this case, a target gene is defined as it has at least one VIS. If a VIS is positioned within several genes (some genes may overlap), all these genes are considered as the target genes. For the HBV, fibronectin 1 (FN1), telomerase reverse transcriptase (TERT1) and lysine‐specific methyltransferase 2B (KMT2B) had the largest number of VISs. Specifically, it was found that 424, 286, and 261 HBV VISs were inserted into these three genes, respectively (Figure 2C), while these genes were also reported as recurrent target genes by HBV integration.^[^ ^12^, ^20^ ^]^ Among the target genes of HPV, Bis(5′‐adenosyl)‐triphosphatase (FHIT), low‐density lipoprotein receptor‐related protein 1B (LRP1B) and DNA repair protein RAD51 homolog 2 (RAD51B) were integrated more frequently than other genes, with the number of VISs reaching 54, 36, and 36, respectively. Previous studies have indicated that VIS tends to occur near the transcript start site (TSS).^[^ ^15^ ^]^ Accordingly, the distance between VISs and their nearest TSSs was estimated for HBV and HPV (Figure 2E). Only about 10% of VISs were found to be within the 10 kb regions of TSSs. When the range was expanded, ≈70% VISs were located within 50 kb flanking the TSS (including TSS). We further analyzed the closest CpG islands (CGIs) within the flanking region of the VISs (Figure 2F). Our result revealed that ∼5% of VISs were inserted in the 10 kb regions of CGIs. Even when the region was extended to 50 kb, the proportion was still less than 50%.

### 2.2 Predicting Oncogenic Viral Integration Sites by DeepVISP

**Figure 3:**
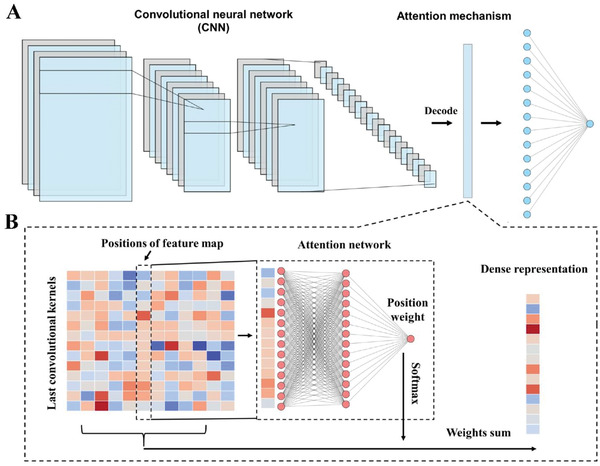
The deep learning framework implemented in DeepVISP. A) The overview of deep learning framework in DeepVISP. B) The schematic view of attention architecture.

**Figure 4:**
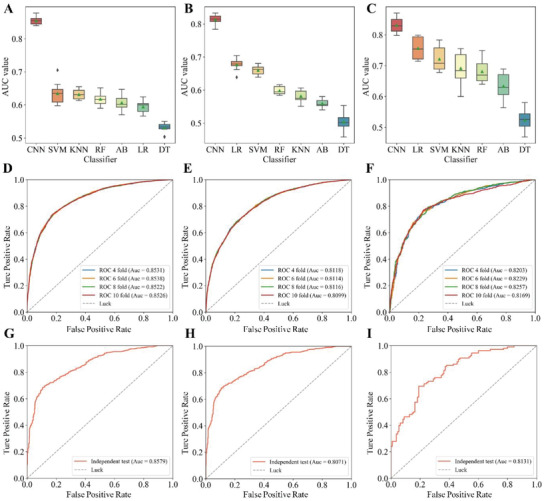
DeepVISP performance evaluation and comparison with other six machine learning methods. Comparison of AUC values between CNN and other six conventional machine learning classifiers, AB, DT, KNN, LR, RF and SVM using A) HBV, B) HPV, and C) EBV data. The abbreviation of methods is provided in the main text. ROC curves and AUC values of DeepVISP using the training datasets with multiple cross‐validations (CVs) in D) HBV, E) HPV, and F) EBV. ROC curves and AUC values of DeepVISP using the independent datasets with multiple CVs in G) HBV, H) HPV, and I) EBV.

We developed a deep learning‐based predictor, namely DeepVISP, for accurately predicting oncogenic VISs in the human genome by automatically learning informative features and essential genomic positions. We tested it using three benchmark datasets we collected. The DNA sequences were encoded and taken as input. Then, two convolution‐pooling modules were followed to make feature extraction and representation. Moreover, an attention layer was joined to link the last convolution‐pooling module and the output layer (**Figure** **3**). In addition to deep learning model, we implemented six other conventional machine learning models, i.e., AB, DT, KNN, LR, RF, and SVM, and compared the performance of DeepVISP with them. The results showed that DeepVISP could achieve moderate to large AUC value improvement (an increase AUC value by 8.43–34.33%) when compared to conventional models in these three viruses. Accordingly, the accuracy of DeepVISP is superior to other conventional models, (**Figure** **4**), indicating that deep learning could effectively extract features for predicting oncogenic VISs.

To further evaluated the accuracy and robustness of DeepVISP, we performed 4‐, 6‐, 8‐, and 10‐fold CVs on the training data sets for each virus and the receiver operating characteristic (ROC) curves are shown in Figure 4. We found that DeepVISP had high performance with the average AUC values of multiple CVs greater than 0.8 in all viruses, with a range from 0.8112 (HPV) to 0.8529 (HBV). For HBV, the AUC values of 4‐, 6‐, 8‐, and 10‐fold CVs were 0.8531, 0.8538, 0.8522, and 0.8526, respectively. The high and consistent AUC values by different CVs in our results demonstrated the promising accuracy and robustness of DeepVISP models. Moreover, using the independent datasets, we tested the adaptability of our models. As shown in Figure 4, DeepVISP achieved AUC values of 0.8579, 0.8071, and 0.8131 in HBV, HPV, and EBV, respectively, indicating that DeepVISP can provide accurate prediction on independent dataset.

### 2.3 DeepVISP Decoded Regulatory Factors Involved in Oncogenic Viral Integration

The convolutional step represents the engine of the CNN framework and highly attributed to the performance of the model. The kernels of the convolution layer generated several weight matrices over the inputs to distinguish important patterns. Numerous studies have utilized kernels of the first convolutional layer to derive motifs from massive sequence data. In the DeepVISP, multiple convolution kernels were applied to detect representative motifs within the input DNA sequences. We calculated the positions of the maximum activation from the output vectors of convolutional layer and mapped them to the input sequences to extract a number of subsequences. Only those subsequences whose maximum activation score exceeds a certain threshold (i.e., the maximum of the MMAs per class) were considered. For each kernel, all the extracted subsequences were aligned to create a PWM format. We measured the importance of detected motifs and calculated the score of each motif. As a result, a total of 248, 211, 245 informative motifs were characterized for HBV, HPV, and EBV, respectively. All motifs and the distribution of the PWMs were graphically illustrated (available at https://bioinfo.uth.edu/DeepVISP/Download.php). For instance, the top three motifs for HBV integration were “AAAAATA,” “ATTATAAA,” and “TAGAAAGT” with scores of 0.083, 0.076, and 0.068. And the most representative motifs for HPV integration were “ATCCTTTA,” “ACATTTTG,” and “CTATAATA.”

**Figure 5:**
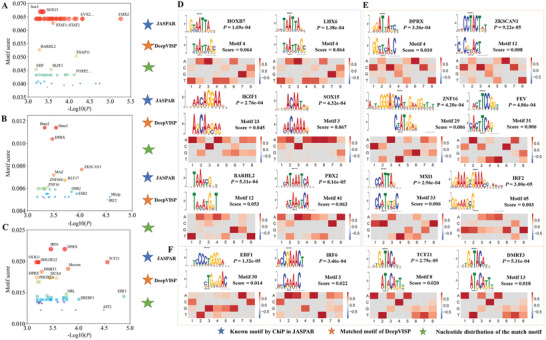
Top 50 informative motifs learned from DeepVISP and also matching the known regulatory motifs in A) HBV, B) HPV, and C) EBV using the TOMTOM server. *P* < = 0.01 was used as the statistical threshold. It also displays the sequence logos and distributions of the position weighted matrices (PWMs) learned by DeepVISP and the matching DNA‐binding transcription factors in JASPAR2020 database for D) HBV, E) HPV, and F) EBV.

After interacting with their host cells, viruses generally dominated the expression of host RNA by virally encoded molecules, which can be realized through physical interactions between a viral transcriptional co‐factor and a host transcription factor affecting the downstream host gene expression.^[^ ^21^ ^]^ Accordingly, we used TOMTOM^[^ ^22^ ^]^ to compare the motifs that DeepVISP learned for different oncogenic viruses with the known DNA motifs in the JASPAR2020,^[^ ^23^ ^]^ a database of transcription factor binding profiles. We identified a good number of regulatory factors whose binding site preference was highly consistent with the motifs reported by DeepVISP. More specifically, among the top 50 learned PWMs of HBV, HPV, and EBV integrations, 13, 16, and 18 could be matched with known motifs of 60, 30, and 45 DNA‐binding transcription factors, respectively. (**Figure** **5**A–C).

Specifically, the PWM M4 of HBV matched DNA motif of homeobox protein Hox‐B7 (HOXB7) (Figure 5D), which acts as a sequence‐specific transcription factor associated with cell proliferation and differentiation. Many studies have shown that HOXB7 plays an important role in the pathogenesis and progression of liver cancer.^[^ ^17^ ^]^ For instance, by activating the MAPK/ERK pathway induced by the basic fibroblast growth factor (bFGF), Wang et al. found that HOXB7 increased the proliferation, migration, and invasion of HCC cells, whereas its expression was highly correlated with poor prognosis and tumor recurrence of HCC patients^[^ ^17c^ ^]^. Moreover, the PWM M4 of HBV was consistent with the motif of the DNA‐binding protein LIM/homeobox protein Lhx6 (LHX6) (Figure 5D). It has been reported that expression of *LHX6* gene was significantly down‐regulated by DNA methylation and plays a tumor suppressing role during hepatocarcinogenesis, suggesting that *LHX6* can be considered as a potential target gene and a biomarker for liver cancer treatment.^[^ ^18^ ^]^ In another example, the PWM M23 of HBV could match the motif of DNA‐binding protein Ikaros (IKZF1). A previous study demonstrated that abnormal function of the IKZF1/MYC/MDIG axis affected the progression of liver cancer by regulating H3K9me3/p21 activity.^[^ ^19^ ^]^ We also carefully examined the motifs learned for HPV (Figure 5E). For example, the PWM M45 of HPV was similar to the motif of the DNA‐binding protein of interferon regulatory factor 2 (IRF2). The IRF family proteins regulate viral and cellular gene expression involving many extracellular signals, and they are involved in cell immunity and oncogenesis. A previous study suggested that *IRF2* participated in early transcription of HPV‐16 gene and was associated with the regulation of cell growth.^[^ ^24^ ^]^ Moreover, PWM M29 of HPV matched the motif of the zinc finger protein 16 (ZNF16). *ZNF16* is a tumor associated gene and its alteration might promote malignancy of tongue squamous cell carcinoma (TSCC) cells.^[^ ^25^ ^]^ We also found that the PWM M30 of EBV matched the motif of the transcriptional activator, namely, transcription factor COE1 (EBF1) (Figure 5F). Interestingly, *EBF1* is the direct target gene of Epstein‐Barr virus nuclear antigen 1 (EBNA1) and is involved in EBV‐infected B‐lymphocyte survival.^[^ ^26^ ^]^ Other DNA‐binding transcription factors corresponding to the PWMs learnt by DeepVISP are summarized in Tables S4–S6 in the Supporting Information. Although it remains unclear how these binding elements present in the human genome associated with oncogenic virus latency and pathogenesis, our results provided potential guidance to probe *cis*‐regulatory factors in the human genome for future study of their oncogenic viral integration and clinical outcome.

### 2.4 Sequence Patterns for Oncogenic Viral Integration

**Figure 6:**
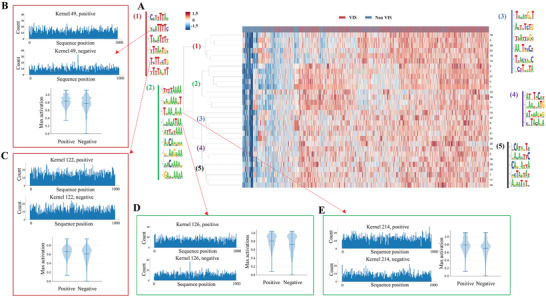
Cluster analysis of the DeepVISP motifs for HBV viral integration. A) Five clusters were identified from the top 30 kernels of DeepVISP with all training data as input and the distribution of co‐occurring motifs in different clusters. B,C) Two representative motifs in cluster 1. D,E) Two representative motifs in cluster 2. The histogram shows the positions of the maximum activation, i.e., the positions where the motif was extracted from the DNA sequences. The violin plots display the distribution of the maximum activation values for HBV VISs and the randomly selected non‐VIS DNA segments.

Deep neural network can capture strong motifs multiple times. These motifs are called dominant ones, and the dominant patterns may be interrelated. Based on the maximum activations calculated for each sequence‐kernel pair, we performed the hierarchical clustering analysis to explore extensive intercorrelated sequence patterns for oncogenic viral integration. Taking HBV as an example, five clusters were identified among the top 30 kernels of CNN model. **Figure** **6**A shows five clusters and the distribution of the correlated motifs. In this figure, the column is the input DNA sequence, and the row represents the kernel. There were six motifs in cluster 1 and most of these motifs contained two consensus k‐mers of “TTT” or “TTTT,” which was strongly clustered in the positive samples. We further analyzed two motifs, e.g., “TCATTTTC” and “TCTTTCT,” in this cluster (Figure 6B,C). Histogram plots show the position of the maximum activations where the motif was extracted from the DNA sequences. The violin plots show the distribution of the maximum activation values for VISs and randomly selected non‐*VDSs*. Here, VDS refers **V**IS‐centric **D**NA **s**egment (see Methods). In addition to the occurrence in the same cluster, we observed that both motifs had higher activation scores and larger distribution in the positive samples than non‐*VDSs*. Additionally, cluster 2 consisted of eight similar motifs. This cluster was modeled by a group of predominant k‐mers, including “AA,” “AAA,” and “AAA.” A great number of k‐mers cognate in the kernels allowed the model to capture the full complexity of viral integration specificities and the effect of the flanking bases, leading to a richer representation. We checked two motifs in this cluster, “AAAAATAA” and “ATTATAAA” (Figure 6D,E). Both motifs held higher proportions and activation scores in positive samples than non‐*VDSs*. The results above indicated that the dominant k‐mers in the simultaneous motifs might contribute to the classification performance of the CNN models and play an important guidance role in viral recognition of host genes. Neural networks are always hard to interpret and require detours to supplement information. Nevertheless, clustering may provide some approximative guidance on the sequence preference of oncogenic viral integration.

### 2.5 Online Server for the VIS Prediction

**Figure 7:**
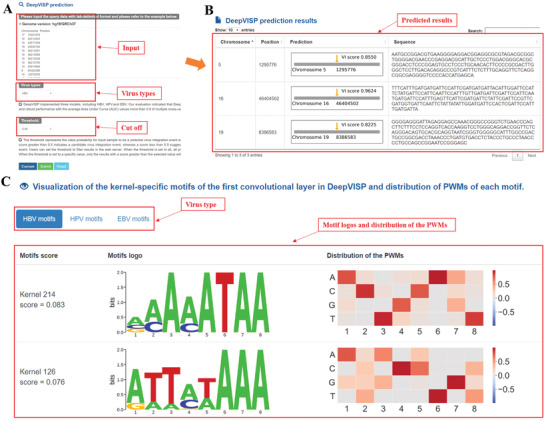
DeepVISP online web server. A) Users can input the query data with tab‐delimited format in the textbox and choose the three virus types for prediction: HBV, HPV and EBV. To control false‐positive rates, a set of threshold values are listed (i.e., 0.7, 0.75, 0.8, 0.85, 0.9, 0.95, and 0.99). For a specific threshold value, only the sites whose scores are greater than the threshold value will be displayed. B) The webpage for the predicted results. All generated prediction results are saved in a tabular format containing detailed information regarding the positions of predicted VISs, scores, and DNA sequences around the predicted VISs. (C) The browse webpage showing all the motifs learned by DeepVISP (only showing two here) and the distributions of the PWMs for each motif.

An online DeepVISP web server is implemented and is publicly available at https://bioinfo.uth.edu/DeepVISP. The web server is developed on the open‐source web platform LAMP (Linux‐Apache‐MySQL‐PHP) and has been tested on popular web browsers, including Google Chrome, Internet Explorer, Safari and Mozilla Firefox. On the DeepVISP prediction web page (**Figure** **7**A), users can input the query data with tab‐delimited format in the textbox. The first column of the query sequence represents the chromosome number, and the second column is for position. Users can choose different types of viruses for prediction. To control false‐positive rate, a set of threshold values is made available (e.g., 0.7, 0.75, 0.8, 0.85, 0.9, 0.95, 0.99). The threshold value here refers to the class probability of the input sequencing being a potential VIS. A score above 0.5 indicates a candidate virus integration event. Users can set the threshold to filter results in the web server. When the threshold is set to all, all prediction results will be displayed. Users are recommended to select the predicted VISs with higher scores if they will perform further experimental verification. All prediction results generated are stored in a tabular format with detailed information regarding the positions of predicted VISs, scores and DNA sequence around the predicted VISs (Figure 7B). On the browse webpage, it provides a visualization of the motifs that DeepVISP learns and the distributions of the PWMs for each motif (Figure 7C). Users can select different types of viruses to view the motifs, which are sorted by scores.

## 3 Discussion

Virus infection, including its integration into the human genome, is one common factor leading to the development of disease. Although many experiments have generated various datasets to explore oncogenic viral integration, our understanding of the underlying mechanisms remains limited. In addition, it is both labor intensive and costly to generate such experimental data, while computational prediction can be a complementary approach for virus infection and VIS detection. Of note, virus research has become urgently needed because of frequent pandemic events during the recent years, especially the COVID‐19 pandemic this year. The significance of virus infection research has been recognized by the recent announcement of the Nobel Prize in Physiology or Medicine 2020 being awarded to Drs. Harvey J. Alter, Michael Houghton and Charles M. Rice for their discovery of hepatitis C virus. The integration‐associated virus latency has increasingly attracted research interest. In this study, using the benchmark datasets that we manually curated, we first examined the distribution of oncogenic VISs on human chromosomes and pinpointed the genes with high VISs. We further explored some critical features of gene function, such as transcript start sites and CpG islands. Next, we introduced a new deep learning model with attention architecture, named DeepVISP, to accurately predict three types of oncogenic VISs (i.e., HBV, HPV, and EBV) in the human genome. Through automatically extracting informatic features and important genomic positions from the primary DNA sequences with CNN framework, DeepVISP achieved excellent performance: its AUC values were consistently higher than 0.8 for all three oncogenic viruses in all CVs we have evaluated. Even we applied 2‐fold CV, which is rarely used in literature due to the requirement of large dataset, we could have AUC values around 0.8 (HBV: 0.8146; HPV: 0.7571; EBV: 0.7786). When compared with traditional machine learning methods, DeepVISP obtained superior accuracy with the improvement of AUC value from 8.43% to 34.33% in three viruses. In summary, these comparison results demonstrated the robustness and superiority of DeepVISP.

CNN represents a useful approach for motif discovery. Through uncovering sequence motifs enriched in the first convolution layer, we decoded several regulatory factors, such as HOXB7, IKZF1, and LHX6, that might contribute to oncogenic viral integration selection. With the calculated maximum activations for sequence‐kernel pairs, we performed the hierarchical clustering analysis. The results indicated that the dominant k‐mers in some cluster contributed to the classification performance of the CNN model and played an important role in virus recognition of host genes. DeepVISP can be useful in several ways. If a virus is integrated into cellular genome at specific positions that disrupt critically important gene(s), the affected cell may become dysfunctional (e.g., immune system cells) or over‐functional (e.g., cancerous cells). Thus, insertional mutagenesis is a potential risk that may accompany virus integration events. Deep learning methods like DeepVISP can efficiently screen insertional mutagenesis in the large genomic datasets to detect the potential virus infection and specific genes and biological pathways being affected. This will assist with the early detection of oncogenic virus infection and development of clinical gene therapy. Moreover, we can combine the experimental and computational work to save the cost in an effort on effectively searching the potential virus infection sites in the host genomes. To better use our method and data, an online public web server for DeepVISP was implemented at https://bioinfo.uth.edu/DeepVISP.

Our study demonstrates the usefulness of deep learning, especially convolutional neural network, in prioritizing and analyzing oncogenic viral integration. However, the number of VISs in some viruses is not very large, which may affect the classification performance and subsequent motif mining. We expect more functional important and clinically relevant VISs to be discovered, as genome sequencing has become feasible in a typical research lab. Accordingly, a large number of experimental VISs will greatly enhance the prediction power of deep learning model. In addition, more features, such as structural information, gene expression and survival information, should be considered when such data on oncogenic viral integration are available. While a considerable number of VISs has been identified, the biological or regulatory roles of most of these sites remain unknown. It is particularly important to explore the interplay between the known virus insertion features and the functional elements in the host genome. We anticipate that our DeepVISP method will help researchers to find candidates for experimental validation or future discovery. Such VISs will provide more valuable insights for the future studies of oncogenic viral integration in the human cells.

## 4 Experimental Section

##### Data Collection and Processing

The experimental VIS data was retrieved from the in‐house VISDB database.^[^ ^15^ ^]^ It had 20588 HBV, 5118 HPV, and 1112 EBV VISs. They were considered as three positive data sets in this work. To train the tumor‐specific models, only retained VISs were retained in the tumor samples. To learn features from surrounding sequence, each VIS was represented by a 1000‐nucleotide **V**IS‐centric **D**NA **s**egment (*VDS*), with 500‐bp flanking regions of upstream and downstream of the VIS (*500*, *500*), respectively. Next, highly similar sequences were removed to ensure the reliability of the benchmark dataset. The CD‐HIT software^[^ ^27^ ^]^ was used to calculate the similarity score and excluded those *VDSs* (*500*, *500*) that had the sequence similarity of more than 90% with each other. After elimination of homologous sequences, three non‐redundant datasets with 4738 HBV, 3459 HPV, and 1089 EBV *VDSs* were retained. Negative data was selected with the size that matched the number of positive samples ten times (i.e., the above non‐redundant VDSs) from a large number of randomly selected non‐VIS containing DNA segments (non*‐VDSs*) (1000 bp in length), followed by eliminating highly similar sequences. Samples were taken out with a 1:1 ratio of positive and negative labels each time for training and calculated the average performance of all models. To facilitate the training and testing of the model, benchmark data sets were strictly separated into non‐overlapping training and testing datasets. These collected benchmark datasets can be downloaded at: https://bioinfo.uth.edu/DeepVISP/Download.php.

##### Feature Encoding

The one‐hot encoding represents the position‐specific composition of the nucleotides in a *VDS*, of which a 4‐digit binary vector s associated with each nucleotide. Specifically, A is encoded by (1, 0, 0, 0), C is encoded by (0, 1, 0, 0), G is encoded by (0, 0, 1, 0) and T is encoded by (0, 0, 0, 1), respectively. For each *VDS (500, 500)*, a 4‐channel input data is encoded as:

(1) $$\begin{array}{ccc} B & = & \left(b_{n1},b_{n2},b_{n3},b_{n4},\;\ldots ,\;b_{n1000}\right),\;b\in \left\{\begin{array}{c} A:\;1,0,0,0\\ C:\;0,1,0,0\\ G:\;0,0,1,0\\ T:\;0,0,0,1 \end{array}\right.,\\ n & \in & \left\{A,\;C,\;G,T\right\} \end{array}$$

##### Conventional Machine Learning Methods

In this study, six conventional machine learning classifiers, included AB, DT, KNN, LR, RF, and SVM, were implemented. Each classification algorithm was trained using the one‐hot encoding with feature selection by chi‐square test. The receiver operating characteristic curve (ROC) curves were drawn and AUC values were calculated based on the 10‐fold CV to evaluate each algorithm's performance. This process was repeated ten times to ensure the reliability of the results. Moreover, hyperparameter optimization was preformed using the RandomizedSearchCV of scikit‐learn v0.21.3 (https://scikit‐learn.org/) for each classification algorithm to obtain the best model. For the process of hyperparameter tuning, 10‐fold cross validation approach was applied to train the model; that is, the available training data was divided into 10 partitions, while 10 identical models were instantiated. Each model was trained on 9 partitions and then evaluated on the remaining, non‐overlap partition. The verification score of the final model was equal to the average of the 10 verification scores. In this process, the remaining partition does not participate in the training process, and the performance of the model can be notarized. Through this method, the performance of each set of parameters was evaluated, and the corresponding AUC values were calculated. The group of parameters with the highest AUC values was selected as the final parameters of the model. Three measurements of sensitivity (*Sn*), specificity (*Sp*), and Matthews Correlation Coefficient (*MCC*) were calculated to evaluate the prediction performance. The three measurements were defined as below

(2) $$Sn\;=\frac{TP}{TP+FN}\;\;Sp\;=\frac{TN}{TN+FP}\;$$

(3) $$\begin{array}{c} MCC=\frac{\left(TP\times TN\right)\;-\;\left(FN\times FP\right)}{\sqrt{\left(TP\;+\;FN\right)\;\times \;\left(TN\;+\;FP\right)\;\times \;\left(TP\;+\;FP\right)\;\times \;\left(TN\;+\;FN\right)}}. \end{array}$$

##### Deep Convolutional Neural Networks (CNN) with Attention Architecture

In this work, the CNN model was designed with four components, including an input layer, two convolution‐pooling modules, an attention layer and an output layer (Figure 3A). For the input data, each nucleotide in the *VDS (500, 500)* was converted to a binary vector of length 4 by one‐hot encoding (*b_n1_, b_n2_, b_n3_*, …, *b_n1000_*), where *b_n_* stands for the nucleotide at the *n* ^th^ position. In the convolutional layer, different fitters and kernel sizes were adopted over the 4‐channel input data to execute convolution operations and extract the features for different viruses. Each convolution operation refers to a weight matrix (i.e., kernel) that can be considered as a position weight matrix (PWM). More specifically, given a *VDS (500, 500)*, i.e., *B* = (*b_n1_, b_n2_, b_n3_*, …, *b_n1000_*), the convolutional layer calculates *C* = conv(*E*)

(4) $$C_{i,\;j}=\;\sum_{a\;=\;0}^{b-1}\sum_{c\;=\;1}^{4}W_{i,a,c}E_{c,j+a}$$

Where 1 ≤ *j* ≤ 1000 – *b* +1, 1 ≤ *i* ≤ *d*, *b* denotes the kernel size, *d* denotes the kernel number and *W* denotes the kernel weight. The rectified linear unit (*ReLU*) was used as the activation function:

(5) $$ReLU\left(x\right)=\left\{\begin{array}{cc} x, & \mathrm{if}\;\;x\geq 0\\ 0, & \mathrm{if}\;\;x<0 \end{array}\right.$$

where *x* denotes the weighted sum of a neuron. The function of max‐pooling was used to conduct dimension reduction after the convolution and activation.

To better retain the implied sequence characteristic of the *VDS (500, 500)*, an attention layer was introduced into the model^[^ ^16^, ^28^ ^]^ (Figure 3B). The attention layer takes the feature representation of the last convolution‐pooling module as input and calculates a score (i.e., *C*), suggesting whether the neural network should pay more attention to the features at that position. More specifically, column *b* in the feature matrix *F* (*d* × *h* feature matrix) can be considered as a vector (i.e., *v_b_*) that illustrates the features of the *b* ^th^ position in the *VDS (500, 500)*, of which each dimension refers to a kernel of the convolutional layer. Then, the columns of the feature matrix *F* were averaged by using the normalized importance scores *w_b_* as weights, generating a dense feature representation *F^w^*

(6) $$F^{w}=\;\sum_{b\;=\;1}^{h}w_{b}v_{b}$$

(7) $$w_{b}=\;\frac{\exp\left(C_{b}\right)}{\sum_{m\;=\;1}^{h}\exp\left(C_{m}\right)}$$

where *W_b_* denotes the corresponding normalized score and *C_b_* denotes the importance score.

Subsequently, the feature vectors captured by the last convolution‐pooling module and the attention scores were integrated and fed to a logistic regression classifier to acquire an output score that indicates the probability of VIS, which can be defined as follow

(8) $$\mathrm{prediction}\left(y\right)=\frac{1}{1+e^{-y}}\;$$

where *y* denotes the input of the sigmoid node from the combination of convolution‐pooling feature vectors and attention scores. The prediction score ranges between 0 and 1, representing the probability of a *VDS (500, 500)* for a viral integration site.

The hyperparameters of DeepVISP were optimized for each virus with the tree‐structured parzen estimator approach using the Hyperas package. Specifically, 100 evaluations were executed using separate training (inner loop) and validation sets (outer loop). The performance of each set of parameters was evaluated and the corresponding AUC values were calculated. The group of parameters with the highest AUC values was selected as the final parameters of the model. For model training, NVIDIA Tensor Cores with four Tesla V100 were used. The Keras version 2.3, a highly useful neural networks API, and the tensorflow‐gpu 1.15 version were adopted for a rapid parallel computing.

##### Motif Decoding, Comparison, and Clustering

To visualize the motifs learned by DeepVISP, the method described in the previous study was used.^[^ ^29^ ^]^ Given an input sequence, a kernel in the first convolutional layer generates an output vector (i.e., activations). The maximum activation positions were computed and mapped to the input sequence and a subsequence with kernel length can be extracted from the input sequence. Only subsequences whose maximum activation score exceeds the threshold [maximum of the mean maximum activations (MMAs) per class] were taken into account. In this way, it generates a good number of subsequences that can be used to construct a motif. For each kernel, all subsequences extracted were aligned to create a PWM in the MEME motif format.^[^ ^22^ ^]^ The pysster package^[^ ^29b^ ^]^ (https://github.com/budach/pysster) was used for sequence logo generation and the TOMTOM webserver^[^ ^22^ ^]^ (http://meme‐suite.org/tools/tomtom) was used for PWM comparison with JASPAR database,^[^ ^23^ ^]^ which contains a curated, non‐redundant set of profiles derived from published and experimentally defined transcription factor binding sites.

The motif score was calculated as maximum of the MMAs in the positive data minus minimum of the MMAs in the negative data. Accordingly, the score measures the degree of difference across the classes, i.e., kernels that were strongly enriched in some classes but were very weak or even none in other classes. A higher score means that the corresponding kernel is more important for the network to deliver correct predictions. Given the maximum activations for each sequence‐kernel pair, a hierarchical clustering using Ward's method and the Euclidean distance^[^ ^30^ ^]^ was performed and the values were standardized before clustering.

##### Implementation of the Web Service

The online service of DeepVISP was constructed with PHP and JavaScript with a user‐friendly interface. Users can choose three viruses (e.g., HBV, HPV and EBV) and various threshold options. By default, an “All” option was implemented to show all the predictions on VISs. DeepVISP was extensively tested on various web browsers including Google Chrome, Internet Explorer, Safari and Mozilla Firefox. It provides a robust and publicly available service at https://bioinfo.uth.edu/DeepVISP.

## Conflict of Interest

The authors declare no conflict of interest.

## Supporting information

Supporting Information

Supplemental Table 1

Supplemental Table 2

Supplemental Table 3

Supplemental Table 4

Supplemental Table 5

Supplemental Table 6

## Data Availability

Data available in article supplementary material.
