# Supplementary material for: DeepVISP: Deep Learning for Virus Site Integration Prediction and Motif Discovery
Source: Adv Sci (Weinh). 2021 Mar 8;8(9):2004958. doi: 10.1002/advs.202004958 (PMC8097320; doi:10.1002/advs.202004958)
Supplement: Supplementary file 1 — Supporting Information [file ADVS-8-2004958-s006.pdf]

© 2021 Wiley-VCH GmbH

Supporting Information

for *Adv. Sci.*, DOI: 10.1002/advs.202004958

DeepVISP: Deep learning for virus site integration prediction and motif discovery

*Haodong Xu, Peilin Jia, Zhongming Zhao**

Supplementary data for "DeepVISP: Deep learning for virus site integration prediction and motif discovery"

Supplementary Table S1. Full list of virus integration sites of hepatitis B virus (HBV).

Supplementary Table S2. Full list of virus integration sites of human herpesvirus (HPV).

Supplementary Table S3. Full list of virus integration sites of Epstein-Barr virus (EBV).

Supplementary Table S4. List of DNA-binding transcription factors matching the PWMs learnt by DeepVISP for the HBV integration.

Supplementary Table S5. List of DNA-binding transcription factors matching the PWMs learnt by DeepVISP for the HPV integration.

Supplementary Table S6. List of DNA-binding transcription factors matching the PWMs learnt by DeepVISP for the EBV integration.
